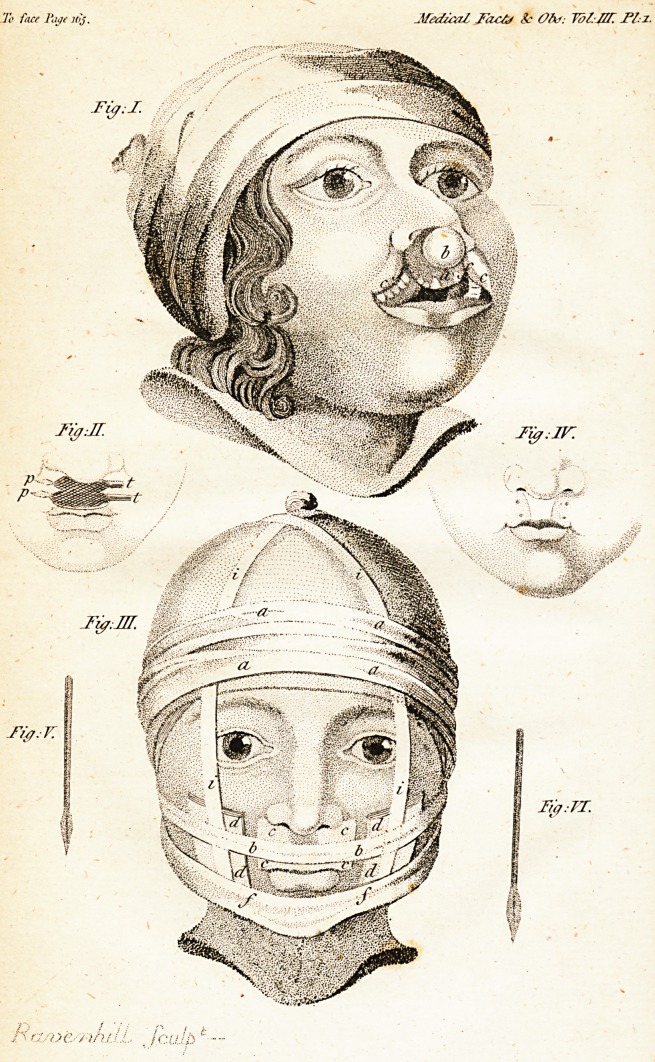# Account of a Case of Double Hare Lip, Accompanied with a Fissure of the Palate; with Remarks

**Published:** 1792

**Authors:** M. Chorin

**Affiliations:** One of the Surgeons of the Hotel Dieu at Paris.


XV. *Account of a Caſe of double Hare Lip, accompanied with a Fiſſure of the Palate; with Remarks. By M.* Chorin, *one of the Surgeons of the Hotel Dieu at Paris.*—Vide *Journal de Chirurgie*, Tom I. 8vo. Paris, 1791.

THE patient, whoſe caſe is here related, was a healthy girl, five years old, who was admitted into the Hotel Dieu at Paris, on the 7th of September, 1790, for the cure of a hare lip, the appearances of which are repreſented in fig. 1. of the annexed plate. In the upper lip, under the noſtrils, there were two fiſſures, one third of an inch in width, which extended into the *foſſæ naſales*, and were ſeparated from each other by a ſmall roundiſh protuberance (b. fig. 1.) connected with the noſe, and ſhorter than either of the two portions of the divided lip. Behind this protuberance was a portion of the upper jaw, half an inch wide, which projected more forwards than the reſt of the maxillary bones, from which it was ſeparated on each ſide by a fiſſure of about a quarter of an inch wide. This bony projection, which at its lower part was on a level with the alveolar proceſs, ſupported the two middle inciſors, (which were ſmaller

ſmaller than uſual, and moveable in their ſockets) and at its upper part correſponded with the *ſeptum narium*, near the lower edge of which was a fiſſure, three quarters of an inch wide, which divided the roof of the mouth and the *velum palati* from before backwards.

The patient could take hold of her food only with the canine teeth and the bicuſpides, ſo that ſhe chewed it with difficulty, and when ſhe attempted to ſwallow, a part of the aliment was forced into the *foſſæ naſales*, and another part came out through the fiſſures in the lips. Experience, however, it ſeems, had taught her to leſſen, in ſome degree, theſe inconveniences, by taking into her mouth only a very little food at a time; and M. Chorin remarks, that ſhe could ſwallow liquids with much leſs difficulty, as by inclining her head a little backwards ſhe was able to pour them, in ſome meaſure, immediately into the pharynx.

With reſpect to her powers of ſpeech, it is obſerved that all the ſounds ſhe uttered were naſal, or, as is commonly ſaid, through the noſe; and that ſhe was able to pronounce vowels pretty diſtinctly, but that her articulation of conſonants was ſuch, that ſhe could be underſtood derſtood only by perſons who were much accuſtomed to her.

In order to bring the protuberance already deſcribed to a level with the lip, and to depreſs the projecting portion of the maxillary bones, M. Deſault, who, as the principal ſurgeon of the Hotel Dieu, undertook the treatment of the caſe, had recourſe to a linen bandage, which paſſed over the upper lip and was fixed at the back part of the neck. The good effects of this bandage in compreſſing the parts in queſtion were ſo obvious, that its uſe was continued till the 18th of September, when the operation was performed.

As the patient was in good health, no other preparation was deemed neceſſary than that of regulating her diet for ſome days previouſly to the operation. M. Chorin has thought it right to obſerve, however, that before this was performed care was taken that her hair ſhould be well combed, and likewiſe that mercurial ointment ſhould be applied to it, in order that, by preventing any uneaſineſs to the patient from vermin, the bandage might be more likely to remain the neceſſary time undiſturbed. At the ſame time lint was placed behind the ears, and in the cavities formed by the cartilages of the ears, ears, that the preſſure of the bandage might be more conveniently ſupported by the patient.

During the time of the operation the patient was ſeated on a high chair, with her head againſt the breaſt of an aſſiſtant, whoſe hands, applied to the cheeks, puſhed the fiſſures of the lips forwards, and at the ſame time compreſſed the external maxillary arteries at the part where they paſs on the lower jaw. M. Deſault, who placed himſelf before the patient, and a little to the right, began the operation by taking hold of the edge of the left portion of the lip with the thumb and fore finger of his left hand, and then with a pair of ſciſſars, rounded on both ſides, and very ſharp, cut out the whole of the red part up to the openings in the noſe, perpendicularly to the thickneſs of the lip, taking care to remove a ſomewhat larger proportion of the lower part where the edge was rounded (c. fig. 1.). He next took hold of the lower part of the protuberance (b. fig. 1.), and, ſtretching it, cut away the left edge of it with the ſame precautions he had uſed with reſpect to the lip. After this he removed, in a ſimilar manner, the right edge of the protuberance and the portion of lip that correſponded with it. He now held the angle of the wound, correſponding ing with the left fiſſure, between his thumb and fore finger, while he paſſed into the lip, at the diſtance of the twelfth of an inch from its looſe edge, and of a quarter of an inch from the wound, a gold pin (fig. VI.), beſpread with cerate, and, puſhing it backwards and a little upwards, brought it out in the fiſſure. The protuberance (b. fig. I.) being now ſo placed as to be on a level with the lip, the pin was puſhed on through this alſo, at about its middle, and from thence carried in the ſame manner through the right portion of the lip.

While M. Deſault was employed in bringing the parts together by holding the two ends of the pin, an aſſiſtant introduced behind the latter, and before the protuberance (b.) and the lip, a loop of waxed thread, which he drew downwards, in order to keep theſe parts on the ſtretch and in contact. Upon this loop the operator introduced another broader piece of waxed thread, which he carried ſeveral times croſs and round the pin, taking care to bring the thread ſometimes acroſs and at others under the protuberance (b.)

He next introduced a ſecond pin (fig V.) a quarter of an inch above the firſt, and paſſed it through the two portions of the lip and through the intermediate protuberance with the ſame precautions he had uſed with the former one. He likewiſe twiſted a piece of waxed thread about this ſecond pin in the ſame manner as he had done about the other, and afterwards carried the thread alternately from one pin to the other, in the manner of what is called the twiſted ſuture, till he had covered the whole ſurface of the lip, (ſee fig. II.). The ends of the thread were then ſecured by a knot, and the loop which had ſerved to keep the parts in contact, and, on the ſtretch, was cut off as high as poſſible.

M. Default now placed on the cheeks two compreſſes (dd. dd. fig. III.), each of them an inch thick, and extending from the maſſeter muſcle to near the junction of the lips, and from the os malæ to the lower jaw. Theſe compreſſes were preſſed forwards, and ſupported in this poſition by an aſſiſtant. Small compreſſes were likewiſe placed between the extremities of the pins and the ſkin, and the lip was covered with a pledgit of lint, over which was laid a compreſs (cccc.) moiſtened with vegeto-mineral water.

The patient's head, previouſly to the operation, had been covered with a cotton cap, which came came down pretty low; over this a bandage, three ells long, and of the ſame breadth as the lip, was now carried from right to left ſeveral times round the head immediately above the eyebrows, and being faſtened by a pin behind the right ear, and on a level with the upper lip, was brought over the compreſs of the ſame ſide, and from thence under the noſe and over the compreſs of the left ſide to behind the left ear, where it was ſecured by another pin: the remainder of the bandage was carried round the head. In order to prevent the compreſſes and bandage, juſt now deſcribed, from being diſturbed, another ſmaller bandage (ii. ii.) was placed on each ſide. The middle of this paſſed obliquely under the chin, and one of the heads of it was brought over one of the compreſſes, while the other was carried behind the ear of the oppoſite ſide to the top of the head, where they were faſtened, care being taken to fix them to the compreſs on each ſide, and to the firſt bandage, by means of pins. The motion of the lower jaw was prevented by a ſling bandage (ff.), the heads of which croſſed each other at the back part of the head; and the whole was rendered ſtill more ſecure by ſeveral turns turns of a bandage (aa. aa.) carried round the head.

The operation, M. Chorin obſerves, was not long, nor the introduction of the pins very painful. The patient, who was put to bed immediately after it, ſlept during a part of the day, and the next morning ſhe complained of no pain, nor was any tenſion of the parts obſervable. The ſmall compreſs was then removed from the lip, and a freſh one, moiſtened, as before, with vegeto-mineral water, applied in its ſtead. On the third day the patient was allowed to eat ſome panada. On the fourth the pins were withdrawn by the point, their blunt ends having been previouſly cleaned, and ſmeared with cerate. On the fifth the threads fell off, and the reunion and conformation of the parts ſeemed to be perfect, (ſee fig. IV.); and the patient's pronunciation was obſerved to be much more eaſy. On the ſeventh the punctures occaſioned by the pins ſuppurated a little; but on the tenth they were cicatriſed, ſo that the marks of them could hardly be perceived. On the thirty-eighth the patient quitted the Hoſpital. M. Chorin adds, that he has ſince had occaſion to ſee her repeatedly, and has found that ſhe articulates diſtinctly; that the lip lip is of its natural length; that the fiſſure of the palate is diminiſhed one third; and that the alveolar circle is regular.

M. Chorin is aware that ſeveral inſtances of deformity, nearly ſimilar to that which is the ſubject of the preſent caſe, are to be met with in books. The ancients, he obſerves, always conſidered the cure of ſuch caſes as abſolutely impoſſible; while, on the other hand, the moderns, from an opinion that the projection of the middle portion of the maxillary bones is to be conſidered as the greateſt obſtacle to the re-union of the lips, have adviſed it to be cut out: but experience, he contends, has ſhown that it is always eaſy either to bring together the lips over this bony projection, or to reduce the latter, by the preſſure of a bandage, to a level with the lateral parts of the jaw, ſo that its exciſion muſt conſequently be uſeleſs. Beſides, he obſerves, ſuch an operation is liable to many objections: it can hardly fail to occaſion inflammation of the neighbouring parts; it will leave a conſiderable ſpace between the maxillary bones; it will deprive the lip of its point of ſupport at the place where it is di-

 vided; vided; and if the reunion takes place, in ſpite of the diſadvantages of ſuch an arrangement, the action of the muſcles will ſoon leſſen the ſpace between the maxillary bones, and the upper jaw will become contracted enough to fall within the under one, a circumſtance which, at the ſame time that it renders maſtication very difficult, will occaſion a freſh deformity.

With reſpect to the operation of this ſpecies of hare lip, ſurgeons, he obſerves, are of different opinions concerning the manner of performing it, and the inſtrument fitteſt to be employed in it, as well as the means that are moſt likely to procure or facilitate the reunion of the parts. For while ſome have thought to render the operation more ſimple by reuniting one of the ſides of the lip to the middle part, and waiting till this ſhould be completely healed before they proceeded to operate on the other ſide; others have given the preference to the method adopted in the preſent caſe by M. Default, by performing the operation on both ſides at the ſame time.

Although Severinus, in his Treatiſe *de efficaci Medicina*, long ago recommended the uſe of the biſtoury in operations of this ſort, yet there are ſtill, our author obſerves, many ſurgeons who, in theſe caſes, give the preference to to the sciſſars, and, in his opinion, not without reaſon; for with the sciſſars the operation, he contends, is performed more ſpeedily and eaſily: in uſing them the ſurgeon is never under the neceſſity of ſeparating the lip from the gums, becauſe he does not cut upon paſteboard, as with the biſtoury, and he himſelf can hold the part that is to be cut: with the sciſſars alſo the inciſion is more regular than with the biſtoury, the parts with the latter being almoſt always unequally divided; and experience has ſhown that the edges of the wound reunite with the ſame facility and quickneſs after an operation with the sciſſars as when the knife has been employed.

The ſuture, M. Chorin obſerves, was for a long time ſuppoſed to be the only means of obtaining the reunion of a hare lip, and many ſurgeons, he adds, ſtill conſider it as the moſt certain and ſuitable method to be adopted in difficult caſes. The inconveniences which are ſometimes thought to be occaſioned by it oftentimes depend, he is convinced, on the manner of performing it, or on the dreſſings that are made uſe of. A great number of hare lips, ſucceſsfully treated by ſuture at the Hotel Dieu, might be brought, he tells us, in ſupport of

 this this aſſertion. Beſides, the bandage alone, however perfect it may be ſuppoſed to be, can never, he contends, keep the parts together ſo exactly and ſecurely as the ſuture; it cannot prevent the blood and the ſaliva from inſinuating themſelves between the edges of the wound; neither can it lengthen parts that are too ſhort, or aſſiſt in elevating ſuch as are too much depreſſed, advantages, he obſerves, which cannot be denied to the ſuture in the hands of a dexterous and experienced operator.

M. Chorin learns from Heiſter * that ſome German empirics were in the habit of uniting the parts in theſe caſes by ſtrong threads introduced at ſuitable intervals, in the manner of what is called the interrupted ſuture; but the inſufficiency of this method, our author obſerves, has long been known, and ſurgeons have adhered to the uſe of pins in theſe caſes, but have differed much about the compoſition and ſhape of theſe inſtruments. M. Chorin gives the preference to thoſe made of gold, becauſe they are not liable to ruſt, and it is poſſible to render their points as ſharp and nearly as cutting as thoſe of ſteel.

* Inſtitut. Chirurg. 4to. Amſtelædami, 1739, p. 674.

Ravenhill Sculpt.

Speaking of the bandage employed in the cafe which is the fubject of this paper, M. Chorin obferves that it is more fimple than any of the other uniting bandages which have been invented for the fame purpofe; that it acts folely on the compreffes and cheeks; that it lies fmooth and without preffing on the lip, and of courfe does not endanger the cutting of the latter by the pins; and that if the compreffes are properly preffed forwards at the moment it is applied, it will be found to act in the fame manner as another bandage will do, the heads of which are made to crofs each other under the nofe.

### Explanation of Plate I.

Fig. I. State of the patient when admitted into the Hofpital.

- a. projecting portion of the jaw.
- b. the protuberance between the two portions of the divided lip.
- c. c. rounded angles of the divifion of the lip.

Fig. II. The twifted future.

- p. p. points of the pins.

- t. t. their blunt ends.

Fig. III. Shows the apparatus employed in the caſe.

cccc. a ſmall compreſs placed on the wound.

dd. dd. thick compreſſes ſerving to preſs the cheeks forward.

bb. part of the uniting bandage paſſing over the compreſſes of the lips and cheeks.

ii. ii. bandages ſupporting the compreſſes of the cheeks.

ff. the ſling bandage.

aa. aa. turns of the roller fixing the whole of the apparatus.

Fig. IV. State of the lip after the cure.

Fig. V. and VI. Shape of the pins.

## Figures and Tables

**Fig: I. Fig: II. Fig: III. Fig: IV. Fig: V. Fig: VI. f1:**